# Real-world use of tafasitamab preceding CD19-directed chimeric antigen receptor T-cell therapy for relapsed or refractory diffuse large B-cell lymphoma

**DOI:** 10.1186/s40364-024-00706-6

**Published:** 2025-01-23

**Authors:** Narendranath Epperla, Loretta J. Nastoupil, Bruce Feinberg, John Galvin, Prathamesh Pathak, Theresa Amoloja, Danielle Gentile, Kim Saverno

**Affiliations:** 1 Huntsman Cancer Institute, University of Utah, Salt Lake City, UT USA; 2 The University of Texas MD Anderson Cancer Center, Houston, TX USA; 3 Cardinal Health, Dublin, OH USA; 4 Incyte Corporation, Wilmington, DE USA

**Keywords:** CAR-T, CD19, Chimeric antigen receptor T-cell, R/R DLBCL, Real-world study, Tafasitamab

## Abstract

Potential CD19 antigen loss following CD19-directed therapy has raised concerns over sequential use of these therapies. Tafasitamab, a CD19-targeting immunotherapy, combined with lenalidomide, is approved for relapsed or refractory diffuse large B-cell lymphoma (R/R DLBCL) treatment in adults ineligible for autologous stem cell transplantation. This retrospective analysis examined characteristics and outcomes of adults with R/R DLBCL who received tafasitamab preceding CD19-directed chimeric antigen receptor T-cell (CAR-T) therapy in a real-world setting. Nine patients received tafasitamab and lenalidomide immediately preceding CAR-T. Median (first quartile [Q1]–third quartile [Q3]) follow-up time since tafasitamab initiation was 26.1 (18.0–28.0) and after CAR-T was 9.3 (1.9–16.7) months. Of the 9 patients, 4 had complete response, 4 had partial response, and 1 had stable disease following tafasitamab; all discontinued tafasitamab due to disease progression. Median (Q1–Q3) tafasitamab therapy duration was 11.0 (8.1–14.1) months. Three patients had CD19 testing following tafasitamab discontinuation, and all tests were positive. Median (Q1–Q3) time from tafasitamab discontinuation to CD19 testing was 7 (6–9) days. Among the 9 patients, median (Q1–Q3) time from tafasitamab discontinuation to CAR-T administration was 3.2 (2.3–3.6) months. Four patients had complete response, 3 had partial response, and 1 had progressive disease as best response to CAR-T; 1 patient had data unavailable. This small real-world analysis demonstrated disease response to CAR-T therapy and detectable CD19 expression following tafasitamab treatment, adding to literature investigating treatment outcomes associated with sequential use of anti-CD19 therapies in patients with R/R DLBCL.

**Supplementary Information:**

The online version contains supplementary material available at 10.1186/s40364-024-00706-6.

## To the editor

CD19 represents an attractive therapeutic target for the treatment of diffuse large B-cell lymphoma (DLBCL), owing to its early-stage ubiquitous expression on B-cell surface and efficient internalization upon antibody binding [1]. Several therapeutic options targeting CD19 exist for patients with relapsed or refractory (R/R) DLBCL ineligible for autologous stem cell transplant, including tafasitamab, an anti-CD19 monoclonal antibody, loncastuximab tesirine, an antibody-drug conjugate, and CD19-directed chimeric antigen receptor T-cell (CAR-T) therapies (axicabtagene ciloleucel, tisagenlecleucel, and lisocabtagene maraleucel) [1, 2]. It is not uncommon for patients with prior exposure to anti-CD19 therapies to receive subsequent treatment with CD19-targeting agents of a different class [3, 4]. However, sequential use of CD19-directed therapies for R/R DLBCL is complicated by concerns over potential decrease in CD19 antigen availability, either through temporary epitope masking secondary to antibody binding, or antigen escape via CD19 downregulation or genetic alterations [1, 2]. There is no consensus on the optimal sequencing of CD19-directed therapies, as clinical trials of these therapies for R/R DLBCL frequently exclude patients with prior exposure to CD19-targeting agents and there are limited real-world data [2].

We recently conducted a retrospective, multisite, physician-abstracted medical chart review of adults with R/R DLBCL in the United States who received tafasitamab as part of routine care [5]. Herein, we examine the characteristics, treatment patterns, and outcomes of a prespecified subgroup that received CAR-T following tafasitamab therapy; results are summarized using descriptive statistics and detailed methods are reported in Supplementary Appendix.

Of the total of 181 patients included in the real-world study of tafasitamab, 9 patients had received tafasitamab plus lenalidomide as a line of therapy (LOT) immediately preceding CAR-T and all had discontinued tafasitamab due to disease progression; median (first quartile [Q1]–third quartile [Q3]) duration of tafasitamab LOT immediately preceding CAR-T was 11.0 (8.1–14.1) months. All 9 patients were still alive at the latest data collection; median (Q1–Q3) follow-up time since tafasitamab initiation and from CAR-T administration was 26.1 (18.0–28.0) and 9.3 (1.9–16.7) months, respectively. Demographics and clinical characteristics of these 9 patients are presented in Table 1. The majority (*n* = 7; 78%) of these 9 patients received tafasitamab in second-line, followed by CAR-T in third-line.

**Table 1:** Demographics and characteristics of patients who received tafasitamab preceding CAR-T therapy

**Variable**	**Patients receiving tafasitamab preceding CAR-T therapy ** *N* = 9
Sex at birth, male,* n* (%)	3 (33.3)
Age at tafasitamab initiation, years, median (Q1–Q3)	63.3 (57.5–69.0)
Race, *n* (%)	
White	6 (66.7)
Asian	2 (22.2)
American Indian or Alaska Native	1 (11.1)
Ethnicity, *n* (%)	
Hispanic	1 (11.1)
Non-Hispanic	8 (88.9)
ECOG PS at tafasitamab initiation, *n* (%)	
0–1	7 (77.8)
≥ 2	2 (22.2)
Ann Arbor stage at tafasitamab initiation, *n* (%)	
III	1 (11.1)
IV	8 (88.9)
R-IPI score at tafasitamab initiation,* n* (%)	
1–2 (Good prognosis)	2 (22.2)
3–5 (Poor prognosis)	7 (77.8)
Presence of double- or triple-hit B-cell lymphoma, *n *(%)	
Double-hit	2 (22.2)
Triple-hit	0
Negative	4 (44.4)
Unknown	3 (33.3)
Tumor volume at tafasitamab initiation,* n* (%)	
Non-bulky (≤ 7,500 mm)	4 (44.4)
Bulky (> 7,500 mm)	5 (55.6)
DLBCL line of therapy in which tafasitamab and CAR-T were received,^a^ *n *(%)	
Tafasitamab in 2L then CAR-T in 3L	7 (77.8)
Tafasitamab in 3L then CAR-T in 4L	1 (11.1)
Tafasitamab in 4L then CAR-T in 5L	1 (11.1)
Primary refractory,^b^ *n *(%)	2 (22.2)
Received first-line anti-CD20 therapy, *n* (%)	9 (100.0)
Received ASCT prior to tafasitamab, *n* (%)	1 (11.1)

*Abbreviations:* *2L* second-line, *3L* third-line, *4L* fourth-line, *5L* fifth-line, *ASCT* autologous stem cell transplant, *CAR-T* chimeric antigen receptor T-cell, *DLBCL* diffuse large B-cell lymphoma,* ECOG PS* Eastern Cooperative Oncology Group performance status, *Q1* first quartile, *Q3* third quartile, *R-IPI* Revised International Prognostic Index

^a^One line of therapy was considered to have ended when the patient discontinued treatment with a regimen; if a drug was added to the ongoing treatment, this was considered a new line of therapy. If a drug within a combination was held or discontinued, this was not considered a new line of therapy. CAR-T therapy (± bridge therapy) was counted as 1 line of therapy. Additionally, salvage chemotherapy followed by ASCT was considered a single line of therapy

^b^Defined as disease progression while receiving first-line therapy or disease progression occurring ≤ 6 months after the completion of first-line therapy

Best responses to tafasitamab were evaluated based on the 2007 Cheson [6] and 2014 Lugano criteria [7] in 5 and 4 patients, respectively. Complete response (CR) and partial response (PR) to tafasitamab were reported in 4 patients (44%) each and 1 patient (11%) had stable disease; median (Q1–Q3) time to best response was 6.0 (3.9–6.6) months. Overall response rate (ORR) was 89%. Among the 9 patients who received tafasitamab prior to CAR-T, 3 had CD19 tested following tafasitamab discontinuation (immunohistochemistry, *n* = 3; flow cytometry, *n* = 2). All 3 patients had positive CD19 test results; median (Q1–Q3) time from tafasitamab discontinuation to first CD19 testing was 7.0 (6.0–9.0) days.

Treatment and utilization patterns of CAR-T therapy are summarized in Table 2. Of the 9 patients who received CAR-T following tafasitamab discontinuation, 7 received bridge therapy (treatment that typically begins after leukapheresis while awaiting CAR-T manufacturing) between tafasitamab discontinuation and CAR-T administration. Despite progression on tafasitamab in the prior LOT, 3 patients were reported to have received tafasitamab plus lenalidomide as bridge therapy. Best responses to CAR-T were evaluated based on the 2007 Cheson [6] and 2014 Lugano criteria [7] in 4 patients each; 1 patient had no response data available. Four patients (44%) achieved CR and 3 (33%) PR, and 1 (11%) patient experienced progressive disease as their best response to CAR-T. The ORR to CAR-T following tafasitamab treatment was 78%; median (Q1-Q3) time to best response was 2.1 (1.9–3.2) months. There was insufficient follow-up to calculate robust estimates for duration of response to CAR-T among the subgroup of patients who responded to CAR-T therapy following tafasitamab administration, which is a limitation of the study.

**Table 2:** Treatment and utilization patterns of CAR-T therapy in patients who had received prior tafasitamab treatment

**Variable**	**Patients receiving tafasitamab preceding CAR-T therapy** *N* = 9
Distance patient had to travel to receive their CAR-T infusion, *n* (%)	
<30 miles	2 (22.2)
31–60 miles	5 (55.6)
61–120 miles	2 (22.2)
Received CAR-T infusion in an inpatient setting, *n* (%)	9 (100.0)
Bridge therapy received prior to CAR-T^a^, *n *(%)	
Polatuzumab + bendamustine/rituximab	2 (22.2)
Gemcitabine/oxaliplatin/rituximab (R-GemOx)	2 (22.2)
Tafasitamab + lenalidomide^b^	3 (33.3)
No bridge therapy	2 (22.2)
CAR-T infusion received, *n* (%)	
Axicabtagene ciloleucel	8 (88.9)
Tisagenlecleucel	1 (11.1)
Time between referral and CAR-T infusion, months, median (Q1–Q3)	3.5 (2.9–4.2)
Time from apheresis to CAR-T infusion,^c^ days, median (Q1–Q3)	34.0 (28.0–43.0)
Time from tafasitamab discontinuation to CAR-T infusion, months,^d^ median (Q1–Q3)	3.2 (2.3–3.6)

*Abbreviations:* *CAR-T* chimeric antigen receptor T-cell, *LOT* line of therapy, *Q1* first quartile, *Q3* third quartile

^a^Bridge therapy was not defined in the electronic case report form and therefore the therapies and timing associated with bridge therapy were left to the physicians’ discretion

^b^The tafasitamab LOT ended on the date of disease progression and tafasitamab bridge therapy started after this date

^c^Among the 6 patients with apheresis date available

^d^The latter of tafasitamab LOT or bridge therapy

In the absence of high-quality prospective clinical trials to inform the optimal use of sequential CD19-directed therapies for R/R DLBCL, concerns about potential alterations to tumor CD19 expression pattern following anti-CD19 therapy continue to exist [2]. Consistent with other reports of detectable CD19 expression following tafasitamab treatment [8, 9], our preliminary findings of positive CD19 test results in all 3 patients with available test results do not support the notion of epitope masking by tafasitamab reported by some investigators [10], especially given the time between tafasitamab discontinuation and collection for all 3 biopsies were within the 17-day half-life of tafasitamab. However, CD19 test results were only available for 3 of the 9 patients, precluding a more robust assessment of the effects of tafasitamab treatment on CD19 expression. Nonetheless, the observed ORR of 78% *(n* = 7/9) following CAR-T therapy among patients previously treated with tafasitamab is also consistent with the small number of studies showing prior CD19-directed therapy does not limit responses to subsequent treatment with CAR-T [11, 12].

In summary, in this small real-world analysis, patients treated with tafasitamab achieved responses to subsequent CAR-T therapy, and among the few patients with available test results, tumor CD19 expression was detectable following tafasitamab discontinuation. Our findings add to the growing body of literature investigating treatment outcomes associated with sequential anti-CD19 therapies in patients with R/R DLBCL. Future prospective studies with larger sample size are warranted to confirm the findings noted in our study.

## Supplementary Information

Supplementary Material 1.

## Data Availability

All data generated or analyzed during this study are included in this published article (and its supplementary information files).

## Abbreviations

CAR-T: Chimeric antigen receptor T-cell
CR: Complete response
DLBCL: Diffuse large B-cell lymphoma
Q1: First quartile
Q3: Third quartile
LOT: Line of therapy
ORR: Overall response rate
PR: Partial response
R/R: Relapsed or refractory
