# Supplementary material for: Real-world use of tafasitamab preceding CD19-directed chimeric antigen receptor T-cell therapy for relapsed or refractory diffuse large B-cell lymphoma
Source: Biomark Res. 2025 Jan 23;13:18. doi: 10.1186/s40364-024-00706-6 (PMC11755875; doi:10.1186/s40364-024-00706-6)
Supplement: Supplementary file 1 — Supplementary Material 1. [file 40364_2024_706_MOESM1_ESM.pdf]

SUPPLEMENTARY APPENDIX

Real-world use of tafasitamab preceding CD19-directed chimeric antigen receptor T-cell therapy for relapsed or refractory diffuse large B-cell lymphoma

Narendranath Epperla, Loretta J. Nastoupil, Bruce Feinberg, John Galvin, Prathamesh Pathak, Theresa Amoloja, Danielle Gentile and Kim Saverno

Supplemental Methods

Physicians from Cardinal Health's Oncology Provider Extended Network abstracted retrospective demographic and clinical data for eligible patients into electronic case report forms between February 22 and March 29, 2023. Eligible patients initiated tafasitamab (with or without concomitant lenalidomide) on or after October 21, 2020 as part of routine clinical care for the treatment of R/R DLBCL. Patients were required to be at least 18 years of age at time of tafasitamab initiation and have ≥ 4 months of follow-up from tafasitamab initiation unless deceased during follow-up. Additional follow-up data were collected approximately 10 months after initial data collection, between December 18, 2023, and January 31, 2024. Herein, we examine the characteristics, treatment patterns, and outcomes of a prespecified subgroup of patients who received CD19-directed CAR-T therapy following tafasitamab therapy; results are summarized using descriptive statistics. This study adhered to all standard research guidelines and received approval and exemption for obtaining informed consent from patients by a central Institutional Review Board.
